# Alternative Splicing in Human Physiology and Disease

**DOI:** 10.3390/genes13101820

**Published:** 2022-10-08

**Authors:** Pinelopi I. Artemaki, Christos K. Kontos

**Affiliations:** Department of Biochemistry and Molecular Biology, Faculty of Biology, National and Kapodistrian University of Athens, 15701 Athens, Greece

Since the discovery of alternative splicing in the late 1970s, a great number of alternatively spliced transcripts have emerged; this number has exponentially increased with the advances in transcriptomics and massive parallel sequencing technologies. However, several questions remain unanswered regarding the splicing pattern of genes and the role of these transcripts. In this Special Issue, “Alternative Splicing in Human Physiology and Disease”, key aspects of this field, accompanied by formerly unknown perspectives, are analyzed. We have assembled 22 articles from prominent authors in the field, investigating the splicing pattern and potential role of several genes with significant implications in cell fate and thus in physiology and disease.

Alternative splicing is a key regulatory process for the proper function of human cells, and its deregulation has been associated with several pathological states. The review by Liu et al. provides interesting and useful information about the manner in which alternative splice variants affect human physiology by providing specific disease examples. Additionally, they provide up-to-date therapeutic approaches based on adjusting the splicing mechanism [1]. The review by Hasimbegovic et al. constitutes a well-written summary about the available findings on the role of alternative splicing in cardiovascular disease, focusing on atherosclerosis, myocardial infarction, heart failure, dilated cardiomyopathy, and circular RNAs in myocardial infarction [2]. Moreover, the interesting research study by Vancheri et al. investigated the role of two *RECK* transcripts in coronary artery disease, showing their differential expression and proposing a functional role of these splice variants in the progression and development of this disease [3]. It should be mentioned that cardiovascular diseases constitute the leading cause of death among diabetic patients. Even though the molecular mechanism by which diabetes induces vascular dysfunction remains largely unknown, the involvement of alternative splicing has been proposed. The review by Cornelius et al. focuses on the current knowledge of alternative splicing and the roles of these transcripts and the respective encoded protein isoforms within the vasculature. Finally, it discusses potential therapeutic strategies to restore aberrant splicing in these pathological states [4].

The alternative splicing process has also been involved in the development and progression of neurogenerative diseases. The review by Lejman et al. analyzes the implication of alternative splicing in the autosomal recessive neurodegenerative disease spinal muscular atrophy, highlighting the key role of *SMN2* splice variants in this disease. These findings are particularly significant since in most cases this disease develops due to mutations in the *SMN1* gene, which is very similar to *SMN2*, having a single C-T substitution in exon 7. It effectively covers the progress achieved in this field by emphasizing the recent accomplishment for the treatment of spinal muscular atrophy using the alternative splicing mechanism [5]. Additionally, the thorough review by Jakubauskienė et al. summarizes the existing knowledge regarding the implication of alternative splicing in Alzheimer’s and Parkinson’s diseases, while it highlights the possible impact of cellular hypoxic microenvironment on the formation of mRNAs assisting in the development of these neurodegenerative diseases [6]. In the context of the treatment of neurogenerative diseases, mesenchymal stem cells have emerged as a promising solution. However, the distinct alternative splicing patterns in this cell type remain elusive. The research article by Jeong et al. shed light on the biology of mesenchymal stem cells, assisting in their functional understanding and future incorporation in therapeutic applications [7].

Interestingly, alternative splicing plays an important role in the regulation of immune activity and immune cell development through alternative splice variants and their respective protein isoforms, supplementing the function of genes involved in the immune reaction. The current knowledge in this field, accompanied by future perspectives, is thoroughly analyzed in the review by Su and Huang [8]. Due to its role in immune system regulation, the implication of alternative splicing in autoimmune disorders has been investigated as well. More specifically, the research study conducted by Papanikolaou et al. revealed extensive alterations in transcription dynamics reflecting on alternative splicing events in systemic lupus erythematosus patients, uncovering the significance of incorporating alternative splicing analyses in the molecular characterization of complex diseases [9].

Interestingly, alternative splicing can affect human vision and cause vision disorders via the intermixing of two genes with a critical role in vision, *OPN1LW,* and *OPN1MW*, and disrupting the exonic splicing code [10], as shown by Neitz M. and Neitz J. Additionally, the late onset of Fuchs endothelial corneal dystrophy has been associated with the CTG18.1 trinucleotide repeat expansion in the *TCF4* gene encoding this transcription factor. This gene is characterized by the production of 46 known transcripts. The expression levels of these transcripts harboring CTG18.1 expansion were investigated in patients with Fuchs endothelial corneal dystrophy by Westin et al., concluding with some preliminary data regarding the effect of this trinucleotide repeat on the expression of *TCF4* transcripts, which require further investigation [11].

The vital role of alternative splicing in cancer is also analyzed in this Special Issue. Specifically, the review by Mehterov et al. provides a comprehensive summary of the recent findings in the alternative splicing mechanisms in the most common malignancies, highlighting its involvement in anticancer drug resistance. It also presents novel opportunities for the development of targeted therapy against cancer-specific transcripts [12]. Another interesting review analyzing the implication of alternative splicing in cancer is the one by Kim et al. However, this review focuses on Hutchinson–Gilford progeria syndrome and small-cell lung cancer. The authors summarize how the formation of a neomorphic protein complex by protein isoforms encoded by distinct splice variants contribute to the pathogenesis of these diseases and suggest therapeutic strategies directed against these protein isoforms [13]. In malignant states, several cellular processes and mechanisms are deregulated, including autophagy. Thus, alternative splicing of genes that are involved in autophagy adds another layer of complexity to the cell stress response and, consequently, cancer. In the review by Habib et al., the diverse roles of alternative splicing in regulating autophagy and their impact on human disease are adequately summarized [14].

Another interesting RNA type that emerges from intergenic splicing is the chimeric RNAs. They were mainly investigated in malignant states and were characterized as ideal biomarkers and drug targets. However, recent studies have proved their expression in normal cells and tissues as well. Interestingly, Chen et al. experimentally validated 17 chimeric RNAs in normal cell lines using paired-end, next-generation RNA sequencing. Additionally, their expression in different cancer and non-cancer cells, including blood samples from healthy donors, was investigated, uncovering their ubiquitous expression pattern [15].

Another hot research topic is the regulation of splicing, which is coordinated not only by intrinsic factors but also by extracellular ones. An interesting example of splicing regulation is the one achieved by long non-coding RNAs (lncRNAs). These molecules are quite important since they participate in gene expression regulation at multiple levels, including replication, transcription, alternative splicing, and translation. Therefore, dysregulation of antisense lncRNA expression plays a crucial role in several biological processes and is associated with tumor progression, metastasis, and resistance to therapeutic agents. Rothzerg et al. investigated the expression of lncRNAs in osteosarcoma patients and presented a molecular signature consisting of 15 lncRNAs with biomarker and therapeutic potential in this disease [16]. Furthermore, an extracellular regulatory factor of splicing is mechanical stimuli. A recent study by Feng et al. showed that the alternative splicing of the *CCND1* gene encoding cyclin D1 is induced by mechanical stress, and that two splicing factors—namely the serine and arginine rich splicing factor 1 (SRSF1) and the SWI/SNF-related, matrix-associated, actin-dependent regulator of chromatin, subfamily e, member 1 (SMARCE1; also known as BAF57)—are possibly responsible for this process [17]. Moreover, another interesting study, conducted by Kim et al., showed that the retinoblastoma cells present alterations to their transcriptome and proteome profiles after their exposure to a hazardous chemical, bisphenol A. These changes could constitute a novel marker for the detection of various diseases associated with environmental pollutants, such as bisphenol A [18].

In recent years, a distinct splicing mechanism, namely back-splicing, has aroused researchers’ interest. This mechanism generates circular RNAs, a novel RNA type with various roles. Although they were first considered as byproducts of splicing, the recent advances in transcriptomics unraveled their abundant expression and have brought them to the fore. An interesting study by Yan et al. analyzes the role of a well-studied circular RNA, circ-Hipk2, and proposes a novel one in myogenesis [19]. More specifically, its overexpression resulted in the inhibition of myoblast proliferation and promotion of myotube formation. Moreover, it was proposed that this circRNA directly binds to the ribosomal protein Rpl7, an essential 60S pre-ribosomal assembly factor, to inhibit ribosome translation, while its expression was shown to be regulated by the activity of the transcription factor SP1. Overall, these findings highlight the circ-Hipk2 potential in regulating ribosome biogenesis and myogenesis. Since the circRNA field is a relatively new one, there are several aspects of circRNA biology that remain unexplored. The alternative splicing and back-splicing of primary transcripts generating multiple distinct circRNAs is such a topic. In the research article by Papatsirou et al., several novel circRNAs of the *PRMT1* gene are described. Additionally, this article provides some novel aspects regarding circRNA biogenesis [20].

Furthermore, technological advances facilitated the research of alternative splicing mechanisms, particularly with the introduction of bioinformatics. Such an emerging tool is the SpliceAI tool, which is suitable for the in silico identification of splice sites. This tool was used in the study conducted by Ha et al. to evaluate the effects of the *NF1* splicing pattern in neurofibromatosis type 1. It concluded that SpliceAI is a convenient web-based tool and could be helpful in clinical laboratories conducting DNA sequencing of the *NF1* gene [21]. Another advance that revolutionizes research in the field of alternative splicing is third-generation (long-read) sequencing, which permits the sequencing of full-length transcripts without the need for fragmentation and assembly. A recent study, conducted by Boti et al., implemented targeted third-generation sequencing, using nanopore technology, to shed light on the alternative splicing products of the E74-like ETS transcription factor 3 (*ELF3*). This study led to the identification of 25 novel transcripts and 2 novel exons of this gene in several cancer cell lines of different tissues of origin [22].

Overall, we believe that the present Special Issue effective summarizes the current knowledge concerning the implication of alternative splicing in human physiology and pathology, providing not only novel mechanisms via which alternative splicing affects cell fate but also novel transcripts with remarkable potential as biomarkers and therapeutic targets. As aforementioned, alternative splicing is implicated in several cell functions and constitutes the main mechanism by which the cell can enrich its coding and non-coding RNA landscape. This is the main reason why its deregulation affects all these pathological and physiological states, which are extensively analyzed in the aforementioned articles. Even though the current knowledge has been widened via the implementation of novel technologies, further investigation is essential for the elucidation of the role of a great number of novel splice variants.

## References

[1] Liu Q., Fang L., Wu C. (2022). Alternative Splicing and Isoforms: From Mechanisms to Diseases. Genes.

[2] Hasimbegovic E., Schweiger V., Kastner N., Spannbauer A., Traxler D., Lukovic D., Gyongyosi M., Mester-Tonczar J. (2021). Alternative Splicing in Cardiovascular Disease-A Survey of Recent Findings. Genes.

[3] Vancheri C., Morini E., Prandi F.R., Alkhoury E., Celotto R., Romeo F., Novelli G., Amati F. (2021). Two RECK Splice Variants (Long and Short) Are Differentially Expressed in Patients with Stable and Unstable Coronary Artery Disease: A Pilot Study. Genes.

[4] Cornelius V.A., Fulton J.R., Margariti A. (2021). Alternative Splicing: A Key Mediator of Diabetic Vasculopathy. Genes.

[5] Lejman J., Zielinski G., Gawda P., Lejman M. (2021). Alternative Splicing Role in New Therapies of Spinal Muscular Atrophy. Genes.

[6] Jakubauskiene E., Kanopka A. (2021). Alternative Splicing and Hypoxia Puzzle in Alzheimer’s and Parkinson’s Diseases. Genes.

[7] Jeong J.E., Seol B., Kim H.S., Kim J.Y., Cho Y.S. (2021). Exploration of Alternative Splicing Events in Mesenchymal Stem Cells from Human Induced Pluripotent Stem Cells. Genes.

[8] Su Z., Huang D. (2021). Alternative Splicing of Pre-mRNA in the Control of Immune Activity. Genes.

[9] Papanikolaou S., Bertsias G.K., Nikolaou C. (2021). Extensive Changes in Transcription Dynamics Reflected on Alternative Splicing Events in Systemic Lupus Erythematosus Patients. Genes.

[10] Neitz M., Neitz J. (2021). Intermixing the OPN1LW and OPN1MW Genes Disrupts the Exonic Splicing Code Causing an Array of Vision Disorders. Genes.

[11] Westin I.M., Viberg A., Bystrom B., Golovleva I. (2021). Lower Fractions of TCF4 Transcripts Spanning over the CTG18.1 Trinucleotide Repeat in Human Corneal Endothelium. Genes.

[12] Mehterov N., Kazakova M., Sbirkov Y., Vladimirov B., Belev N., Yaneva G., Todorova K., Hayrabedyan S., Sarafian V. (2021). Alternative RNA Splicing-The Trojan Horse of Cancer Cells in Chemotherapy. Genes.

[13] Kim B.H., Woo T.G., Kang S.M., Park S., Park B.J. (2022). Splicing Variants, Protein-Protein Interactions, and Drug Targeting in Hutchinson-Gilford Progeria Syndrome and Small Cell Lung Cancer. Genes.

[14] Habib E., Cook A., Mathavarajah S., Dellaire G. (2021). Adding Some “Splice” to Stress Eating: Autophagy, ESCRT and Alternative Splicing Orchestrate the Cellular Stress Response. Genes.

[15] Chen C., Haddox S., Tang Y., Qin F., Li H. (2021). Landscape of Chimeric RNAs in Non-Cancerous Cells. Genes.

[16] Rothzerg E., Ho X.D., Xu J., Wood D., Martson A., Koks S. (2021). Upregulation of 15 Antisense Long Non-Coding RNAs in Osteosarcoma. Genes.

[17] Feng J., Xu X., Fan X., Yi Q., Tang L. (2021). BAF57/SMARCE1 Interacting with Splicing Factor SRSF1 Regulates Mechanical Stress-Induced Alternative Splicing of Cyclin D1. Genes.

[18] Kim C.H., Kim M.J., Park J., Kim J., Kim J.Y., An M.J., Shin G.S., Lee H.M., Kim J.W. (2021). Bisphenol A Exposure Changes the Transcriptomic and Proteomic Dynamics of Human Retinoblastoma Y79 Cells. Genes.

[19] Yan J., Yang Y., Fan X., Tang Y., Tang Z. (2021). Sp1-Mediated circRNA circHipk2 Regulates Myogenesis by Targeting Ribosomal Protein Rpl7. Genes.

[20] Papatsirou M., Diamantopoulos M.A., Katsaraki K., Kletsas D., Kontos C.K., Scorilas A. (2022). Identification of Novel Circular RNAs of the Human Protein Arginine Methyltransferase 1 (PRMT1) Gene, Expressed in Breast Cancer Cells. Genes.

[21] Ha C., Kim J.W., Jang J.H. (2021). Performance Evaluation of SpliceAI for the Prediction of Splicing of NF1 Variants. Genes.

[22] Boti M.A., Adamopoulos P.G., Tsiakanikas P., Scorilas A. (2021). Nanopore Sequencing Unveils Diverse Transcript Variants of the Epithelial Cell-Specific Transcription Factor Elf-3 in Human Malignancies. Genes.

